# Repeated Winning and Losing Experiences in Chronic Social Conflicts Are Linked to RNA Editing Pattern Difference

**DOI:** 10.3389/fpsyt.2022.896794

**Published:** 2022-05-19

**Authors:** Fu-Xia Ru, Fanzhi Kong, Chun-Yan Ren, Yu-Shan He, Shou-Yue Xia, Yu-Ning Li, Ya-Ping Liang, Jun-Jie Feng, Zhi-Yuan Wei, Jian-Huan Chen

**Affiliations:** ^1^Laboratory of Genomic and Precision Medicine, Wuxi School of Medicine, Jiangnan University, Wuxi, China; ^2^Joint Primate Research Center for Chronic Diseases, Institute of Zoology of Guangdong Academy of Science, Jiangnan University, Wuxi, China; ^3^Jiangnan University Brain Institute, Wuxi, China; ^4^Shantou University Mental Health Center, Shantou University Medical College, Shantou, China

**Keywords:** A-to-I RNA editing, dorsal striatum, mouse models, aggressive behavior, repeated winning and losing

## Abstract

Winner-loser effects influence subsequent agonistic interactions between conspecifics. Previous winning experiences could strengthen future aggression and increase the chance of winning the next agonistic interaction, while previous losing experiences could have the opposite effect. Although the role of A-to-I RNA editing has been recently implicated in chronic social defeat stress and aggressive behavior, it remains to be further elucidated in chronic social conflicts in agonistic interactions, especially in the repeated aggression (winners) and repeated defeat (losers) resulted from these conflicts. In the current study, transcriptome-wide A-to-I RNA editing in the dorsal striatum was investigated in a mouse model of chronic social conflicts, and compared between mice repeatedly winning and losing daily agonistic interactions. Our analysis identified 622 A-to-I RNA editing sites in the mouse dorsal striatum, with 23 to be differentially edited in 22 genes, most of which had been previously associated with neurological, psychiatric, or immune disorders. Among these differential RNA editing (DRE) sites four missense variants were observed in neuroligin 2 (*Nlgn2*), Cdc42 guanine nucleotide exchange factor 9 (Arhgef9) BLCAP apoptosis inducing factor (*Blcap*), and cytoplasmic FMR1 interacting protein 2 (*Cyfip2*), as well as two noncoding RNA sites in small nucleolar RNA host gene 11 (*Snhg11*) and the maternally expressed 3 (*Meg3*) gene. Moreover, significant changes were observed in gene functions and pathways enriched by genes with A-to-I RNA editing in losers and especially winners compared to controls. Our results demonstrate that repeated winning and losing experiences in chronic social conflicts are linked to A-to-I RNA editing pattern difference, underlining its role in the molecular mechanism of agonistic interactions between conspecifics.

## Introduction

Agonistic behavior during social conflicts between conspecifics results in winners and losers. Winner-loser effects could influence future agonistic behavior in such conflicts (1). Previous winning experiences could increase the chance of winning the next agonistic interaction while previous losing experiences could have the opposite effect. Repeated winning experiences of encounters between conspecific male mice could increase the tendency of aggressiveness (2). Aggression is associated with various psychiatric and neurological disorders, such as attention deficit hyperactivity disorders, post-traumatic stress disorders, epilepsy, intellectual disability, autism, and schizophrenia. In contrast, losing is perceived as aversive. Repeated social defeat leads to depressive-like behaviors, including anhedonia, anxiety, and social-avoidance (3). Thereby understanding the biological process involved in repeated winning and losing experiences in chronic social conflicts, could be potentially useful for studies on social psychology as well as psychiatric and neurological disorders.

Recently RNA editing changes have been reported in both neurological and psychiatric disorders (4–6), and are associated with both aggressive behavior and social defeat. Altered adenosine to inosine (A-to-I) editing mediated by adenosine deaminase acting on RNA (ADAR) in 5-hydroxytryptamine receptor 2C (*Htr2c*) (7) was implicated in the amygdala of BALB/c mice with social isolation-induced aggressive behavior (8). RNA editing changes have also been found in chronic social defeat stress in mice (6, 9). However, by far, the role of A-to-I RNA editing remains to be further elucidated in agonistic interactions and repeated winning and losing experiences.

The striatum is a subcortical region integrating social information into coding of reward and social action and is involved in agonistic behavior (10). The striatal function could be compromised by social defeat. Mesostriatal transmission is modified in losers defeated by conspecifics (11). The dorsal striatum region has been reported to regulate movement and cognition, while the ventral striatum region modulates reward and emotion (12).

Therefore, the current study conducted a comprehensive investigation of A-to-I RNA editing in the dorsal striatal transcriptome of male mice with alternative social experiences of agonistic interactions (13) and showed that repeated winning and losing experiences were linked to A-to-I RNA editing pattern difference.

## Materials and Methods

### RNA-Seq Dataset

Raw data of RNA-Seq were downloaded from the European Nucleotide Archive (ENA) of the European Molecular Biology Laboratory (https://www.ebi.ac.uk/ena). The dataset (PRJEB36194) included the dorsal striatum from three groups (*N* = 3 per group) of adult C57BL/6J male mice with alternative social experiences (controls, winners, and losers) formed in agonistic interactions (13).

### Read Mapping and Processing

The sequencing data were then processed as previously described (9). Sequencing read quality was analyzed using FASTQC. Read alignment was performed using RNA STAR (version 2.7.0e) and the mouse genome sequence (UCSC mm10) (14) was used. Multiple-mapped and duplicated reads were removed using SAMtools (version 1.9) (15) with base quality scores recalibrated using GATK (version 4.1.3) (16).

### Variant-Calling and Annotation

Variants were called to identify single nucleotide variations (SNVs) by using VarScan (version 2.4.3) (17), filtered using a bioinformatic pipeline as described in our previous study (9), and annotated using the Ensembl Variant Effect Predictor (VEP) (18). In brief, the SNVs were first filtered as follows: base quality ≥ 25, total sequencing depth ≥ 10, alternative allele depth ≥ 2 and alternative allele frequency (AAF) ≥ 1%. The remaining SNVs that met any of the following criteria were further removed unless annotated as known sites in the REDIportal V2.0 database (19): (1) located in homopolymer runs ≥ 5 nucleotides (nt) or simple repeats; (2) located in the mitochondria; (3) located within 6 nt from splice junctions; (4) located within 1 nt from insertions or deletions; (5) within 4% to the ends of reads; (6) annotated as known variants in the dbSNP database Build 142; (7) more than 90% of all samples had an AAF equal to 100% or between 40% and 60%. High-confidence A-to-I SNVs with editing levels ≥ 1% observed in no less than two samples or annotated as known sites in the REDIportal V2.0 database were eventually kept for subsequent data analysis.

### Enrichment Analysis of Gene Function and Pathways

Enrichment analysis of differentially edited genes using DAVID online prediction tools (https://david.ncifcrf.gov/tools.jsp) (20) and Enrichr (21) (https://maayanlab.cloud/Enrichr/). Items with a false discovery rate (FDR) < 0.05 were considered significant.

### Statistical Analysis

The levels of RNA editing among groups were compared using the ANOVA test, and *post-hoc* analysis for pairwise comparisons was conducted using the *Tukey* test. Frequency data were analyzed using *Fisher*'s exact test. Principal component analysis (PCA) analysis was conducted and further visualized using R (version 3.6.3).

## Results

### A-to-I RNA Editing Sites in the Mouse Striatum

**Figure 1:**
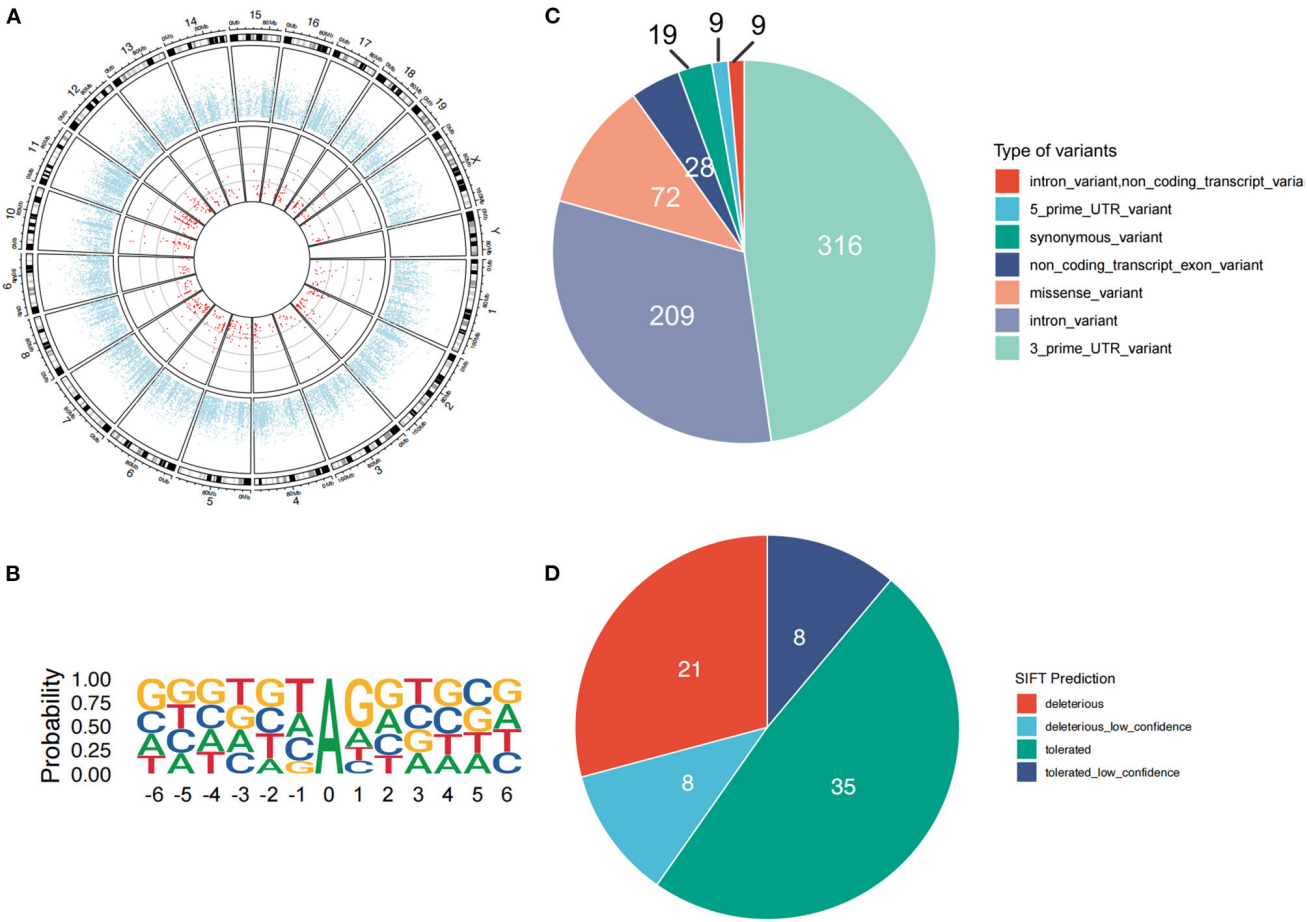
A-to-I RNA editing sites identified from the dorsal striatal transcriptome of male adult mice. **(A)** The red dots denote individual high-confidence editing sites. The blue dots show the mean expression levels of individual genes. **(B)** The motif of sequence context surrounding the A-to-I RNA editing sites. Six nucleotides upstream and downstream of the editing sites are shown. **(C)** Functional categories of various types of the A-to-I RNA editing sites. **(D)** About half of these missense events are predicted by SIFT to possibly be deleterious to the encoded proteins.

In our RNA-Seq data analysis, a total of 662 high-confidence A-to-I RNA editing sites were observed in 342 genes in the mouse striatum (Figure 1A and Supplementary Table 1). These sites, with their editing levels ranging from 1 to 100%, were widely observed across all chromosomes. Motif analysis showed that G was suppressed 1 bp upstream and preferred 1bp downstream the editing sites (Figure 1B). The functional categories of these RNA editing sites included 316 3′-untranslated region (UTR) variants, 209 intronic variants, 72 missense variants, 28 non-coding transcript exonic variants, 19 synonymous variants, 9 5′-UTR variants, and 9 non-coding transcript intronic variants (Figure 1C). SIFT predicted that 29 (40.3%) of the missense variants could have a potential functional impact on the encoded protein (Figure 1D).

### Difference in RNA Editing Patterns Among Alternative Social Experiences

**Figure 2:**
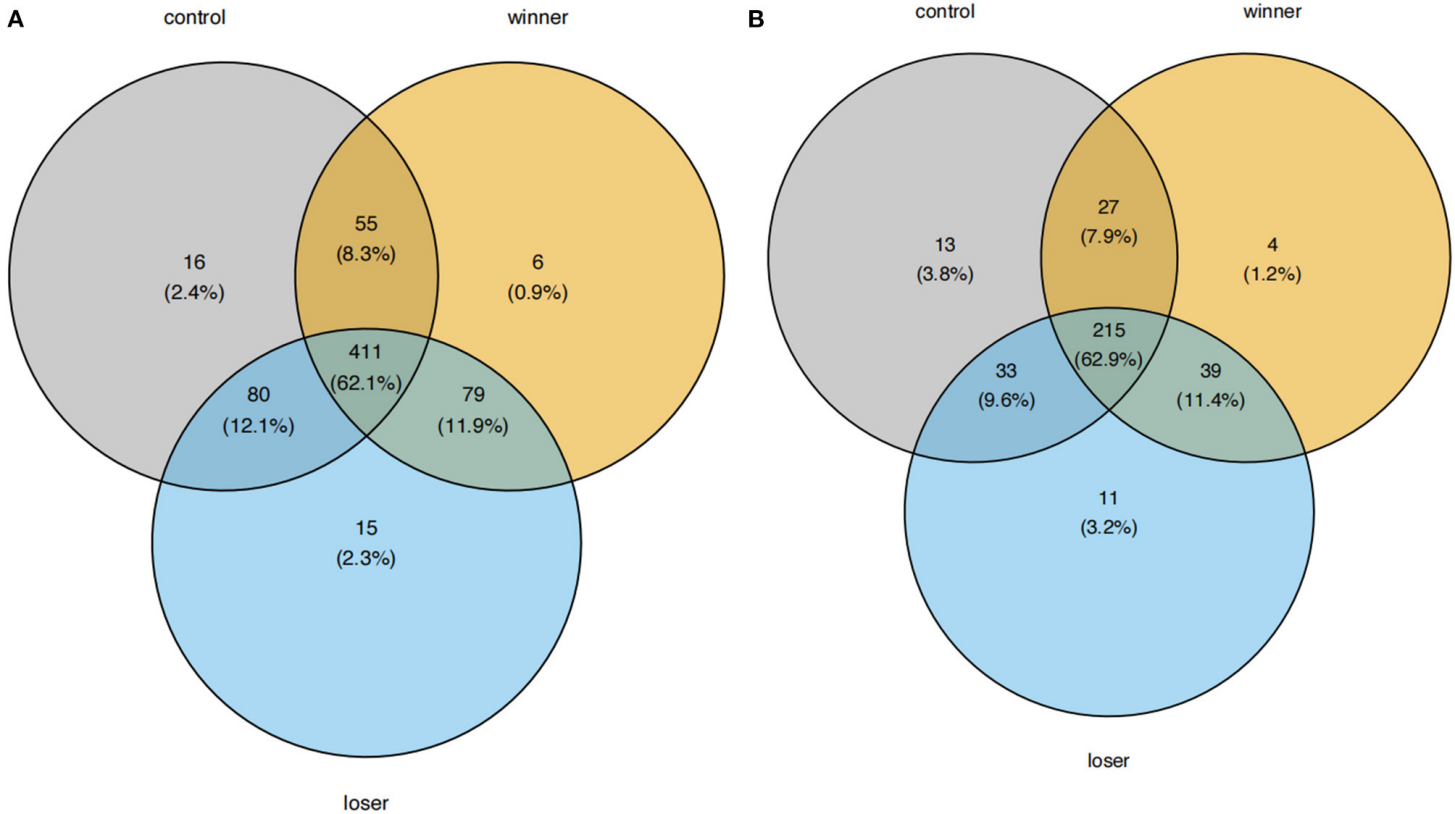
Venn plots showing the comparison of A-to-I RNA editing sites and edited genes among controls, winners, and losers. **(A)** A-to-I RNA editing sites and **(B)** edited genes are shown, respectively.

We then looked into the A-to-I RNA editing sites and edited genes in individual groups. 540, 531, and 577 sites were found in controls, winners, and losers, respectively (Figure 2). Among these RNA editing sites, 411 (62.1%) were shared by all three groups. In addition, 16, 6, and 15 sites were uniquely detected in controls, winners, and losers, respectively. As for edited genes, 275, 273, and 293 were found in controls, winners, and losers, respectively. 215 (62.9%) of these edited genes were shared by all three groups. 13, 4, and 11 genes were uniquely edited in controls, winners, and losers, respectively. No significant difference in the number of editing sites and edited genes were found among the three groups (data not shown).

**Figure 3:**
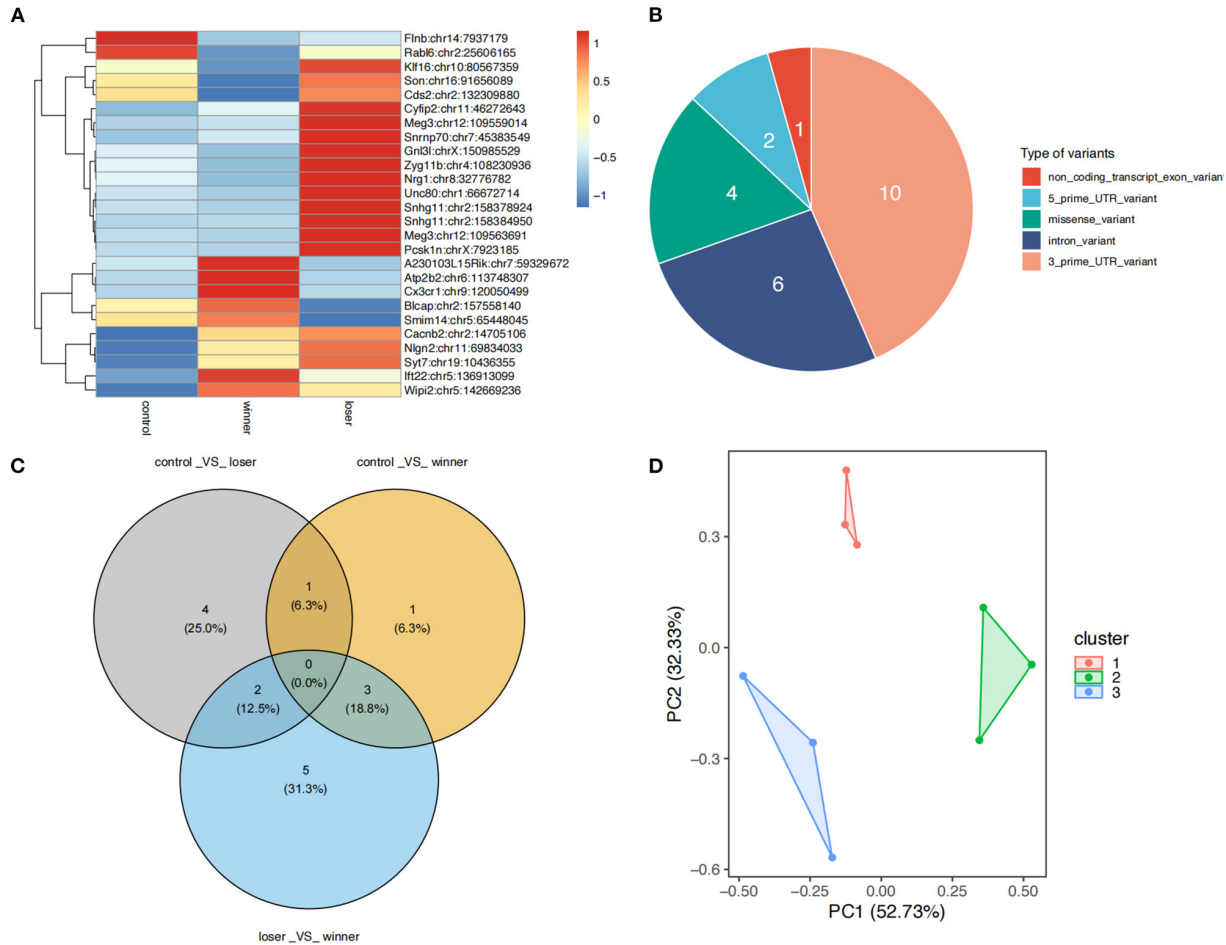
Differential A-to-I RNA editing sites among controls, winners, and losers. **(A)** A total of 23 DRE sites are found. **(B)** Functional categories of various types of DRE sites. **(C)** Principal component analysis of the 23 DRE sites among the three groups. **(D)** Venn plots showing the comparison of DRE sites showing significance in *post-hoc* tests. The *post-hoc* tests are conducted for pairwise comparisons among controls, winners, and losers.

A total of 23 differential RNA editing (DRE) sites in 22 genes were found among the three groups (Figure 3A). DAVID annotation showed that most of these DRE genes were associated with neurological, psychiatric, or immune disorders (Supplementary Table 2). Among these differentially edited sites 10 were located in the 3′-UTR, 6 in introns, 4 in coding regions (missense), 2 in the 5′-UTR, and 1 in non-coding transcript exons (Figure 3B). There were no significant expression changes in most of these differentially edited genes (data not shown). PCA with the 32 differential edited sites showed that the groups of samples clustered separately from each other, with 52.73 and 32.3% contribution from PC1 and PC2 to the total variance (Figure 3C). In addition, *post-hoc* analysis showed that the DRE sites in ten genes including LUC7 Like (*Luc7l)*, Fas activated serine/threonine kinase (*Fastk*), CDP-diacylglycerol synthase 2 (*Cds2*), vesicle associated membrane protein 4 (*Vamp4*), BLCAP apoptosis inducing factor (*Blcap*), Rab-like protein 6 (*Rabl6*), SRC kinase signaling inhibitor 1 (*Srcin1*), small integral membrane protein 14 (*Smim14)*, tyrosine 3-monooxygenase/tryptophan 5-monooxygenase activation protein beta (*Ywhab*), and Neuregulin 1 (*Nrg1*) had significantly different editing levels between winners and losers with opposite change directions compared to controls (Figure 3D and Supplementary Figure 1).

It was noted that among the four missense DRE sites, two ranked the top two among all DRE sites, including p.Asn136Asp in the neuroligin 2 (*Nlgn2*) gene (Nlgn2:chr11:69834033, *P* = 3.0 × 10^−4^) and p.Arg380Gly in the Cdc42 guanine nucleotide exchange factor 9 (*Arhgef9*) gene (Arhgef9:chrX:95058979, *P* = 4.0 × 10^−4^). Compared to controls, winners had an increased editing level of Nlgn2:chr11:69834033, and losers had the highest editing level of Nlgn2:chr11:69834033. The editing of Arhgef9 p.Arg380Gly was observed only in controls but not in winners or losers. Another two missense DRE sites included p.Gln5Arg in *Blcap* (Blcap:chr2:157558140, *P* = 0.019) and p.Lys320Glu in the cytoplasmic FMR1 interacting protein 2 (*Cyfip2*) gene (Cyfip2:chr11:46272643, *P* = 0.019). The editing level of *Blcap* p.Gln5Arg was increased in winners but decreased in losers compared to controls. In contrast, the editing level of *Cyfip2* p.Lys320Glu was only significantly increased in losers.

In addition to protein-coding genes, two long noncoding RNA (lncRNA) genes were also differentially edited, including Small Nucleolar RNA Host Gene 11 (*Snhg11*) (Snhg11:chr2:158378924, *P* = 0.041) and the Maternally Expressed 3 (*Meg3*) gene (Meg3:chr12:109559014, *P* = 0.042).

### Functional Relevance of Altered RNA Editing

Annotation results from DAVID showed that 13 out of the 22 differentially edited genes were associated with neurological or psychiatric disorders: C-X-C motif chemokine ligand 14 (*Cxcl14*), *Cds2, Arhgef9, Fastk, Srcin1, Cyfip2*, kelch like family member 2 (*Klhl2*), kinesin family member 5C (*Kif5c*), muskelin 1 (*Mkln1*), *Nrg1, Nlgn2*, replication protein A1 (*Rpa1*), and *Ywhab* (Supplementary Table 3).

**Figure 4:**
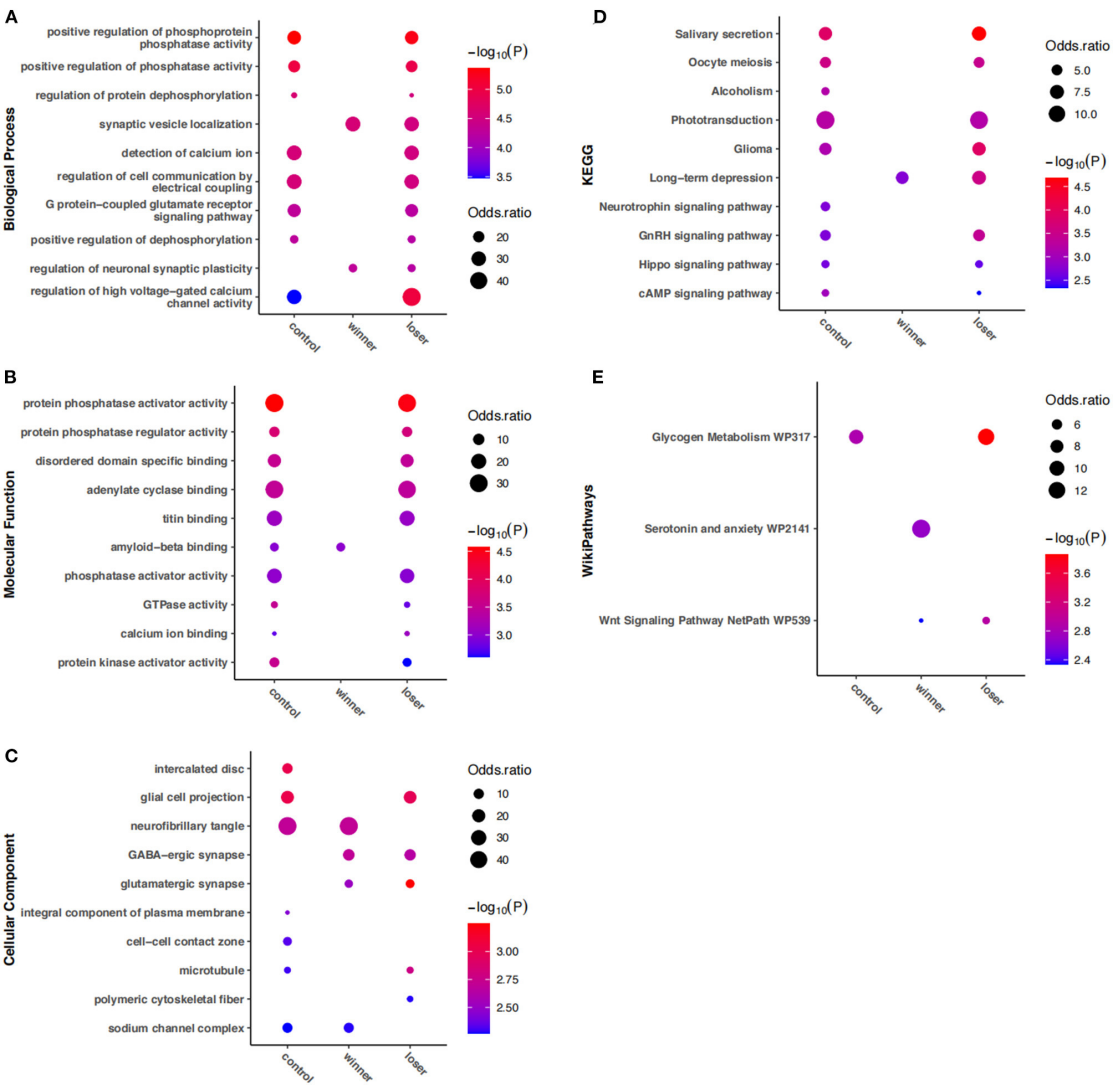
Gene ontology and KEGG pathways enriched by DRE genes among controls, winners, and losers. No more than 10 items with the most significant *P*-values are shown for **(A)** biological processes, **(B)** molecular functions, **(C)** cellular components, **(D)** KEGG pathways, and **(E)** wikipathways, which are insignificant in at least one group.

To get the full picture of the functional relevance of RNA-editing involved in alternative social experiences, enrichment analysis of all edited genes in each group was then compared. Notably, the results in Figure 4 showed most of the top edited gene functions and pathways in the mouse striatum were observed in both controls and losers, but not in winners. The enrichment uniquely lost in winners consisted of biological processes mainly related to G protein–coupled glutamate receptor signaling pathway, regulation of dephosphorylation and high voltage–gated calcium channel activity; molecular functions mainly related to the activity of GTPase and phosphatase regulator, and binding of calcium ion, titin, and adenylate cyclase; cellular components mainly related to glial cell projection and microtubule; and pathways mainly related to salivary secretion, oocyte meiosis, phototransduction, glycogen metabolism, and signaling pathways of cAMP, Hippo, and GnRH. In contrast, the serotonin and anxiety pathway was uniquely enriched in winners. In addition, enrichment uniquely lost in losers included molecular function related to amyloid–beta binding, and cellular components including neurofibrillary tangle, and sodium channel complex.

Apart from these group-specific changes, common changes were also observed. Cellular components of the intercalated disc, cell–cell contact zone, and integral component of the plasma membrane, and pathways related to alcoholism and neurotrophin signaling were enriched in controls but were lost in both winners and losers. Biological processes related to synaptic vesicle localization and regulation of neuronal synaptic plasticity, cellular components of glutamatergic and GABA–ergic synapse, pathways related to long–term depression, and the Wnt signaling pathway were significantly enriched in both winners and losers but not in controls.

### Enrichment of DRE in Genes With Multiple RNA Editing Sites

Our previous study showed enrichment of DRE genes with multiple G-to-A RNA editing. Our results showed that the top 10 genes with multiple RNA editing (Supplementary Table 4) included four DRE genes including *Snhg11, Meg3, Nrg1*, and *Rpa1*. *Fisher's* exact test further demonstrates that genes with two or more editing sites have a 260% increase in the possibility to be differentially edited compared to those with a single detected editing site (OR = 3.6, 95%CI = 1.4–9.8, *P* = 0.002) (Supplementary Table 5).

## Discussion

Adult mice experiencing alternative social conflicts provide a useful model to study repeated winning and losing experiences in chronic social conflicts. Recently, RNA editing has been reported to be involved in defeated stress as well as in aggressive behaviors (6, 8, 9). The current study investigated the transcriptome-wide RNA editing in the dorsal striatum and showed that divergent patterns of RNA editing were associated with winner-loser effects resulting from agonistic encounters in conspecific male mice.

Our analysis revealed the DRE sites in 10 genes as the most divergent between winners and losers. Most of these genes have been reported to play a role in the nerve system or neurological disorders. For example, the *SRCIN1* gene encoding p140Cap/SNIP, a scaffolding protein localized in dendritic spines, was recently reported as a hub for postsynaptic proteins involved in neuropsychiatric disorders (22). Genetic variations in *VAMP4* were previously associated with suicide attempts (23). *Nrg1* encodes a glycoprotein involved in the formation of the neuromuscular junction (24). Its dysregulation has been linked to mental and mood diseases such as schizophrenia (25, 26) and bipolar disorder (27, 28).

Among these DRE sites, there were four missense DRE sites. A missense variant in *Nlgn2* c.406A>G (p.Asn136Asp) was found to be the most differentially edited among mice with alternative social experiences. Winners had an increased editing level of the variant compared to controls, and losers had the highest editing level. *Nlgn2* encoded encodes a transmembrane scaffolding protein that plays a role in cell-cell interactions in both neurons and other cells types and the formation and remodeling of GABAergic synapses by recruiting and clustering synaptic proteins (29). The gene is involved in memory (30) and cognition (31), and has been associated with a variety of neurological and psychiatric disorders such as anxiety, autism (32), and schizophrenia (33). Conditional knockout of *Nlgn2* in the medial prefrontal cortex of adult mice exhibited chronic changes in synaptic inhibition and cognitive impairments, including decreased anxiety-related response, contextual and cued fear conditioning, and social interactions (31). By far the function of the *Nlgn2* c.406A>G variant remains unclear. Although the missense editing variant p.Asn136Asp was predicted to be tolerant to protein function and structure, sequence prediction by Uniprot indicated that it probably abolished a target site of N-glycosylation for Nlgn2, and thus possibly affected the neural transmission (34). Another c.1138A>G (p.Arg380Gly) was found in *Arhgef9*, which encodes a Rho-like GTPase that regulates Cdc42 (35). The Arhgef9 protein also facilitates receptor recruitment in glycinergic and GABAnergic synapses (36). Mutations of *ARHGEF9* have been associated with neurodevelopmental disorders, such as epilepsy, and autism in humans. *Arhgef9* knockout mice showed enhanced anxiety (37). In addition, two evolutionary conserved missense DRE sites were observed in *Blcap* (p.Gln5Arg) and *Cyfip2* (p.Lys320Glu), which were also reported in humans (38, 39). *Blcap* encodes a protein that stimulates apoptosis and reduces cell growth (40). A-to-I RNA editing can occur at three codons at the N-terminus of Blcap, and its alteration may affect the protein function. Our results suggested such RNA editing in *Blcap* might play a role in neural development and disorders. *Cyfip2* was highly expressed in the brain, and its RNA editing of p.Lys320Glu was increased during the brain development (41, 42). *De novo* variants in *CYFIP2* have been reported to cause intellectual disability, seizures, and early-onset epileptic encephalopathy (43). The expression of *CYFIP2* is changed in autism and fragile X syndrome (44). *Cyfip2* mutations in mice have been reported to regulate cocaine response (45) and Fragile X-like behaviors including increased anxiety-like behavior, decreased startle response, and enhanced prepulse inhibition (46).

It was noteworthy that among the DRE genes, two were long intergenic non-coding RNA (lincRNA) genes, the RNA of which were also the most intensively edited targets in the mouse dorsal striatum. An intronic variant c.207-1847A>G in *Snhg11* and an exonic variant n.1836A>G in *Meg3* were significantly up-regulated in socially defeated losers. The *Snhg11* gene belongs to the non-protein-coding multiple snoRNA host gene family, and two snoRNAs are derived from *Snhg11* introns (47). *Snhg11* has recently been reported to play a neuroprotective role in neuronal injury (48). Nevertheless, the role in RNA editing of *Snhg11* and *Meg3* in socially defeated mice (losers) remains unclear. *Meg3* is a maternally expressed imprinted gene that functions as a lncRNA tumor suppressor (49). Recent studies implicated that *Meg3* also plays a substantial role in the nerve system. Tan et al. showed that *Meg3* modulated AMPA Receptor expression in the primary cortical neuron surface (50). The expression of *MEG3* in immune cells was reported to be differentially expressed in drug naïve psychosis patients compared to those treated with risperidone (51). A study using an Alzheimer's disease rat model reported that upregulation of *MEG3* improved cognitive impairment and alleviates neuronal damage through inactivating the PI3K/Akt signaling pathway (52). A recent important study by Royer et al. reported that alterations in the chromatin structure of specific genes such as *PI3K/AKT* in a social fear mouse model could be potentially regulated by *Meg3* (53). The large numbers of A-to-I RNA editing sites in *Snhg11* and *Meg3 and* differential RNA editing in both genes pointed to a potential role of these two lincRNA genes in repeated social defeat.

Moreover, in line with our previous report, genes with multiple editing sites showed a significantly higher possibility of being differentially edited among alternative social experiences. Among the top ten genes with the most editing sites, four exhibited DRE (Supplementary Tables 2, 3). Such findings were in line with a substantial role of RNA editing in the mouse model of winner-loser effects.

Our gene enrichment analysis indicated dramatically down-regulation of RNA editing in specific gene functions and pathways in winners, especially biological processes related to the G protein–coupled glutamate receptor signaling pathway and high voltage–gated calcium channel activity. Aggressive experience in female Syrian hamsters resulted in an increased expression of postsynaptic density, AMPA receptors, and Group I metabotropic glutamate receptors (54). Increased aggression was reported in knock-out mice of genes encoding voltage-gated calcium channels (55, 56). In contrast, the serotonin and anxiety pathway was specifically enriched in winners. Serotonin has been reported to play a pivotal role in anxiety and aggression in mice, and mice deficient in brain serotonin exhibited exaggerated aggression and decreased anxiety (57). The RNA editing of *Htr2c* encoding serotonin receptor 2C was reported to be increased in isolation-induced aggressive behavior of BALB/c mice (8). Apart from these winner-specific changes, common changes were also observed in both winners and losers. Emerging evidence shows that social experience and stress influences neuronal synaptic plasticity (58). The role of Wnt signaling in the brain have been implicated in various neurodevelopmental and neuropsychiatric disorders (59). Therefore, further study is needed to explore the possible role of RNA editing in these gene functions and pathways.

The current study observed dramatic changes in RNA editing in the publicly available transcriptomic data of mouse dorsal striatum from a mouse model of chronic social conflicts. The findings were limited to the original study's sample size, and further validation and functional analysis of the altered RNA editing sites in the mouse model are thus needed in future studies.

In conclusion, the current study identified transcriptome-wide A-to-I RNA editing in the dorsal striatum in a mouse model of agonistic interactions and provided evidence supporting the link between altered RNA editing and repeated winning and losing experiences. Such findings thus warrant further studies on the biological impact of such alterations on brain function and related neuropsychiatric diseases.

## Data Availability Statement

The datasets presented in this study can be found in online repositories. The names of the repository/repositories and accession number(s) can be found in the article/Supplementary Material.

## Ethics Statement

Ethical review and approval was not required for the animal study because ethical review and approval are not applicable for this study.

## Author Contributions

F-XR and FK performed the bioinformatic analysis. Z-YW improved the data analysis pipeline. C-YR, Y-SH, S-YX, Y-NL, Y-PL, and J-JF participated in the data interpretation and discussion. J-HC conceived the project and planned the study. All authors contributed to the final manuscript.

## Funding

This study was supported in part by grants from the National Natural Science Foundation of China (No. 31671311), the National first-class discipline program of Light Industry Technology and Engineering (LITE2018-14), the Six Talent Peak Plan of Jiangsu Province (No. SWYY-127), the Innovative and Entrepreneurial Talents of Jiangsu Province, the Program for High-Level Entrepreneurial and Innovative Talents of Jiangsu Province, Natural Science Foundation of Guangdong Province/Guangdong Basic and Applied Basic Research Foundation (2019A1515012062), Taihu Lake Talent Plan, and Fundamental Research Funds for the Central Universities (JUSRP51712B and JUSRP1901XNC), and Postgraduate Research and Practice Innovation Program of Jiangsu Province (KYCX20_1946).

## Conflict of Interest

The authors declare that the research was conducted in the absence of any commercial or financial relationships that could be construed as a potential conflict of interest.

## Publisher's Note

All claims expressed in this article are solely those of the authors and do not necessarily represent those of their affiliated organizations, or those of the publisher, the editors and the reviewers. Any product that may be evaluated in this article, or claim that may be made by its manufacturer, is not guaranteed or endorsed by the publisher.

## Supplementary Material

Supplementary Figure 1 The 10 most divergent A-to-I (G) DRE sites between winners and losers.
