# Supplementary figures and images for: Repeated Winning and Losing Experiences in Chronic Social Conflicts Are Linked to RNA Editing Pattern Difference
Source: Front Psychiatry. 2022 May 19;13:896794. doi: 10.3389/fpsyt.2022.896794 (PMC9161819; doi:10.3389/fpsyt.2022.896794)

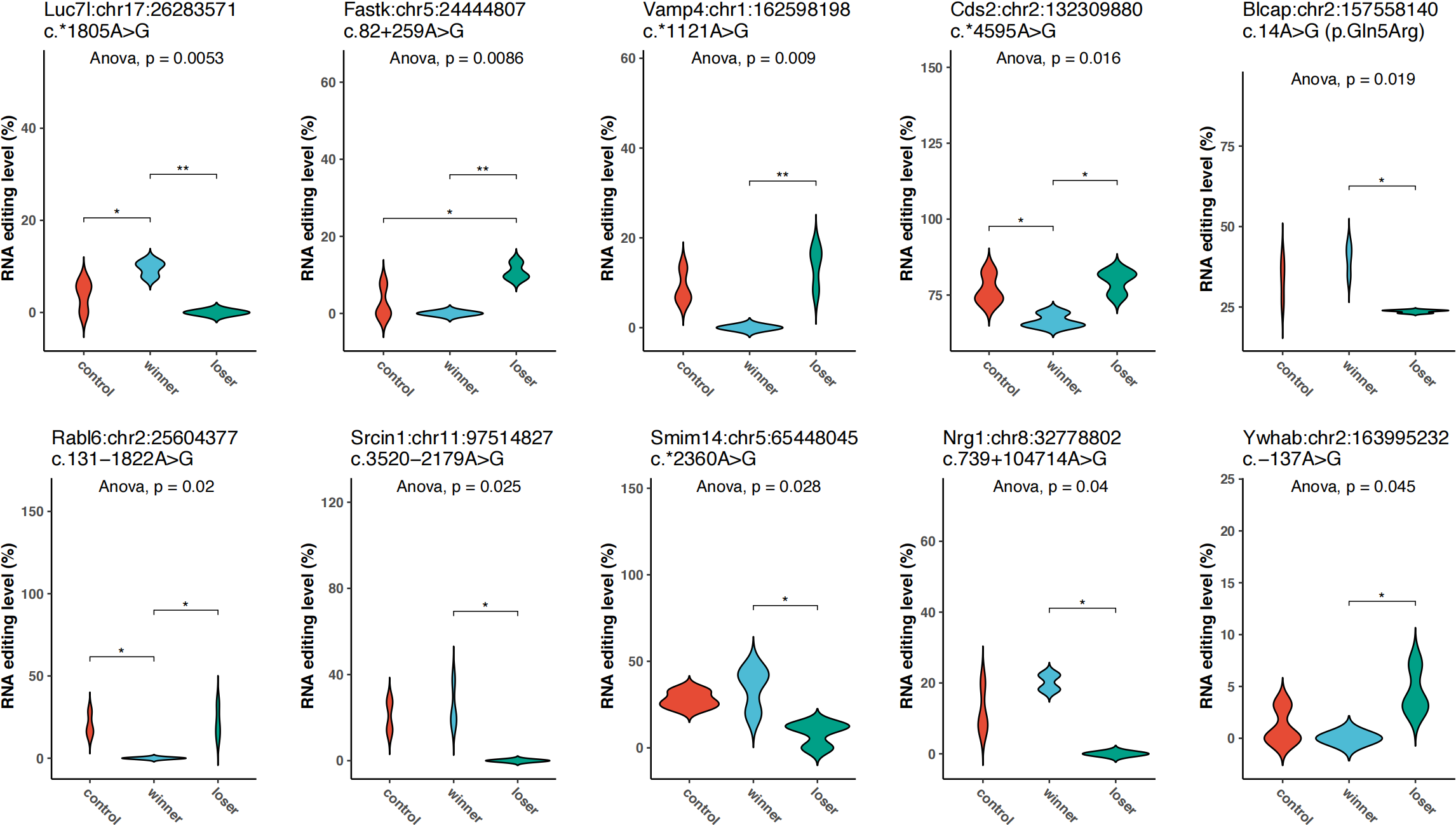

Supplement: Supplementary Figure 1 — The 10 most divergent A-to-I (G) DRE sites between winners and losers. [file Image_1.TIF]
